# Brain region–specific neural activation by low-dose opioid promotes social behavior

**DOI:** 10.1172/jci.insight.182060

**Published:** 2024-12-06

**Authors:** Soichiro Ohnami, Megumi Naito, Haruki Kawase, Momoko Higuchi, Shigeru Hasebe, Keiko Takasu, Ryo Kanemaru, Yuki Azuma, Rei Yokoyama, Takahiro Kochi, Eiji Imado, Takeru Tahara, Yaichiro Kotake, Satoshi Asano, Naoya Oishi, Kazuhiro Takuma, Hitoshi Hashimoto, Koichi Ogawa, Atsushi Nakamura, Hidekuni Yamakawa, Yukio Ago

**Affiliations:** 1 Laboratory for Drug Discovery and Disease Research, Shionogi & Co. Ltd., Toyonaka, Osaka, Japan.; 2 SK Project, Medical Innovation Center, Graduate School of Medicine, Kyoto University, Kyoto, Kyoto, Japan.; 3 Laboratory of Molecular Neuropharmacology, Graduate School of Pharmaceutical Sciences, and; 4 Department of Pharmacology, Graduate School of Dentistry, Osaka University, Suita, Osaka, Japan.; 5 Department of Social Pharmacy, Faculty of Pharmaceutical Sciences, Setsunan University, Hirakata, Osaka, Japan.; 6 Shionogi TechnoAdvance Research Co. Ltd., Toyonaka, Osaka, Japan.; 7 Department of Cellular and Molecular Pharmacology,; 8 Department of Dental Anesthesiology, and; 9 Department of Neurochemistry and Environmental Health Sciences, Graduate School of Biomedical and Health Sciences, Hiroshima University, Hiroshima, Hiroshima, Japan.; 10 Department of Psychiatry, Graduate School of Medicine, Kyoto University, Kyoto, Kyoto, Japan.; 11 Molecular Research Center for Children’s Mental Development, United Graduate School of Child Development, Osaka University, Kanazawa University, Hamamatsu University School of Medicine, Chiba University, and University of Fukui, Suita, Osaka, Japan.; 12 Division of Bioscience, Institute for Datability Science,; 13 Open and Transdisciplinary Research Initiatives, and; 14 Department of Molecular Pharmaceutical Sciences, Graduate School of Medicine, Osaka University, Suita, Osaka, Japan.; 15 Ping An-Shionogi Co. Ltd., Xuhui District, Shanghai, China.; 16 Laboratory of Biopharmaceutics, Graduate School of Pharmaceutical Sciences, and; 17 Global Center for Medical Engineering and Informatics, Osaka University, Suita, Osaka, Japan.

**Keywords:** Neuroscience, Therapeutics, Behavior, Neurodevelopment, Pharmacology

## Abstract

The opioid system plays crucial roles in modulating social behaviors in both humans and animals. However, the pharmacological profiles of opioids regarding social behavior and their therapeutic potential remain unclear. Multiple pharmacological, behavioral, and immunohistological c-Fos mapping approaches were used to characterize the effects of μ-opioid receptor agonists on social behavior and investigate the mechanisms in naive mice and autism spectrum disorder–like (ASD-like) mouse models, such as prenatally valproic acid–treated mice and *Fmr1*-KO mice. Here, we report that low-dose morphine, a μ-opioid receptor agonist, promoted social behavior by selectively activating neurons in prosocial brain regions, including the nucleus accumbens, but not those in the dorsomedial periaqueductal gray (dmPAG), which are only activated by analgesic high-dose morphine. Critically, intra-dmPAG morphine injection counteracted the prosocial effect of low-dose morphine, suggesting that dmPAG neural activation suppresses social behavior. Moreover, buprenorphine, a μ-opioid receptor partial agonist with less abuse liability and a well-established safety profile, ameliorated social behavior deficits in two mouse models recapitulating ASD symptoms by selectively activating prosocial brain regions without dmPAG neural activation. Our findings highlight the therapeutic potential of brain region–specific neural activation induced by low-dose opioids for social behavior deficits in ASD.

## Abstract 2

Non-analgesic low-dose opioid activates pro-social brain regions to ameliorate sociability deficits in ASD models.

## Introduction

Animals including humans and rodents live in a world that is largely socially constructed, where they conduct a wide miscellany of complex social interactions. Social behaviors are essential for the health, survival, and reproduction of animals. Conversely, social behavioral deficits are key features of several neuropsychiatric disorders, especially autism spectrum disorder (ASD). ASD is a neurodevelopmental disorder defined by social communication impairments and restricted, repetitive behaviors (1, 2). Despite the global increase in the ASD burden and the growing demand for effective medications (3), there is currently no approved drug for the core symptoms of ASD ASD; rather, there are only drugs for its comorbidities, such as irritability.

Neuropeptides, such as oxytocin and arginine vasopressin, play a central role in social function. Several molecules targeting oxytocin and vasopressin signaling have been developed in clinical trials for ASD therapeutics. However, they all failed to demonstrate clinically meaningful improvement of social function in patients with ASD (4, 5). The reasons for the failures include (a) a substantial disparity in these pathways between humans and rodents and (b) the optimal levels of oxytocin and vasopressin signaling are unknown.

The opioid system plays a critical role in social behavior as well as pain sensation. In particular, the crucial role of μ-opioid receptors (MORs) in social function has been revealed by genetic and pharmacological studies. For instance, deletions and duplications of genomic regions covering the *OPRM1* gene were identified in patients with ASD (6), and *Oprm1*-mutant mice showed ASD-like symptoms, including sociability deficits and repetitive behaviors (7). However, the optimal level and even directionality (activation/inhibition) of MOR signaling regulation to promote social behavior are controversial (8–11) and have yet to be elucidated, particularly under the pathological condition of ASD.

Here, we aim to elucidate the effects of a wide range of doses of morphine and buprenorphine on social behavior and pain sensation in naive and ASD-like model mice. Moreover, we performed c-Fos expression mapping across the brain regions involved in social behavior to obtain mechanistic insights into the roles of each region in the pharmacological effects of MOR agonists. Thus, we report that only low doses of MOR agonists without an analgesic effect promoted social behaviors in mice by specifically activating prosocial brain regions, such as the nucleus accumbens (NAc). These doses did not activate the dorsomedial periaqueductal gray (dmPAG), which was only activated by high doses of MOR agonists, resulting in inhibition of social behavior. Nonanalgesic low doses of MOR agonists could be potential therapeutic options for sociability deficits in ASD.

## Results

### Low-dose morphine promotes social behavior in naive and valproic acid model mice.

**Figure 1:**
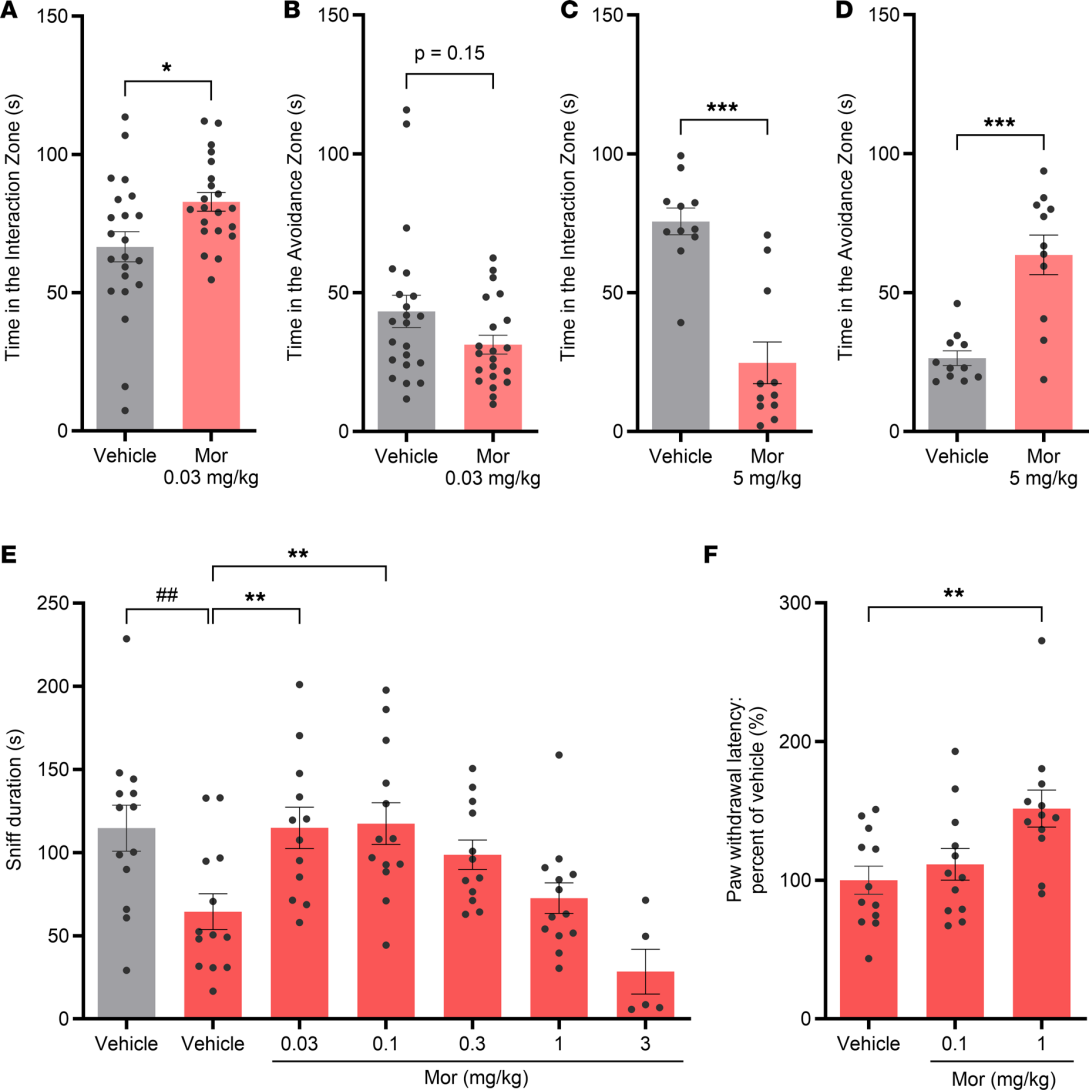
Morphine increased social behaviors in mice only at low doses. (**A**–**D**) Effects of systemic administration of morphine on social behaviors in the single-chamber social interaction test (SIT). Single-housed C57BL/6J mice were s.c. injected with morphine (0.03 or 5 mg/kg) or vehicle at 12–13 weeks of age. The time spent in the interaction zone (**A** and **C**) and the avoidance zone (**B** and **D**) during a 3-minute test period with an unfamiliar mouse kept in a cage (target session) is shown. *n* = 21–22 (**A** and **B**) and 11 animals (**C** and **D**). (**E**) Effects of systemic administration of morphine on social interaction deficits in mice prenatally exposed to valproic acid (VPA) in the reciprocal SIT. VPA (500 mg/kg) or saline was intraperitoneally injected into pregnant CD-1 mice at embryonic day 12.5. Male offspring at 8 weeks of age were s.c. injected with morphine (0.03, 0.1, 0.3, 1, 3 mg/kg) or vehicle, and the duration of sniffing was measured during a 20-minute test period. *n* = 5–13 animals. (**F**) Effects of morphine on thermal nociception in prenatally VPA-treated mice. The withdrawal latency at 49°C was measured with the hot-plate test at 1 hour after administration of morphine (0.1, 1 mg/kg, s.c.). *n* = 12 animals. Data are shown as the mean ± SEM. *P < 0.05, **P < 0.01, ***P < 0.001, by the parametric tests (**A**, **D**, and **E**) or nonparametric ones (**B**, **C**, and **F**) compared with vehicle-treated mice. ^##^*P* < 0.01 compared with mice prenatally exposed to saline (control). Mor, morphine.

First, we assessed the effects of systemic morphine administration on social behaviors of naive C57BL/6J (B6) mice using the single-chamber social interaction test (SIT). Morphine (0.03 mg/kg, s.c. administration) significantly increased the time spent in the interaction zone when an unfamiliar B6 mouse was present (Figure 1A), whereas it did not change the time spent in the empty session without an unfamiliar mouse (Supplemental Figure 2, A and B; supplemental material available online with this article; https://doi.org/10.1172/jci.insight.182060DS1). The time spent in the avoidance zone tended to be decreased by the administration of 0.03 mg/kg morphine (*P* = 0.15 compared with vehicle-treated animals: Figure 1B), whereas such a trend was not observed in the empty session. In contrast, 5 mg/kg morphine significantly decreased the time spent in the interaction zone and increased that in the avoidance zone (Figure 1, C and D). In the empty session, 5 mg/kg morphine decreased the time spent in the interaction zone but did not change the time spent in the avoidance zone (Supplemental Figure 2, D and E). High doses of morphine have been reported to induce hyperlocomotion (12), which could affect the readouts in SIT. Therefore, we examined the effect of morphine on locomotor activity (Supplemental Figure 3A) and found that only 5 mg/kg morphine significantly increased locomotor activity. While hyperlocomotion could contribute to the decrease in time spent in the interaction zone in SIT, it would be unlikely that hyperlocomotion would lead to an increase in time spent in the avoidance zone. Taken together with a greater decrease in time spent in the interaction zone in the target session compared with that spent in the empty session, 5 mg/kg morphine would, at least to some extent, suppress the sociability of the mice. Thus, systemic morphine administration enhanced social interaction behaviors only when a low dose was administered, and that effect was inverted with a higher dose.

Next, we examined the effect of morphine on social interaction deficits in mice prenatally exposed to valproic acid (VPA), which are commonly used as an animal model of ASD (13–16), based on the evidence that fetal valproate exposure is associated with an increased risk of autism and ASDs in humans (17, 18). VPA model was reported to show social behavior deficit in reciprocal SIT but not in the chamber-based SIT without direct physical interactions (19). Therefore, we evaluated the effect of morphine on social behavior of the VPA model using reciprocal SIT. Consistent with results of previous studies, we observed social interaction deficits in VPA-treated mice compared with the behavior of prenatal saline-treated control mice (Figure 1E). These deficits were significantly improved by s.c. administration of lower doses of morphine (0.03 and 0.1 mg/kg), but not with higher doses (0.3, 1, or 3 mg/kg), resulting in an inverted U-shaped dose response (Figure 1E). These findings suggest that systemic administration of low doses of morphine can improve social behaviors in VPA-treated ASD-like animals as well as naive animals (Figure 1, A–D).

To further clarify the dose-response relationship of the effects of morphine on sociability and pain sensitivity, we evaluated pain-related behaviors in mice. Dose-dependent antinociceptive efficacy of morphine in intact mice was detected at doses greater than 1 mg/kg (s.c. administration) (20–24). In VPA-treated mice, we found that 1 mg/kg, but not 0.1 mg/kg, morphine significantly increased the paw-withdrawal latency in the hot-plate test (Figure 1F). These data indicate a difference between the effective doses for suppressing pain behavior and promoting social behavior.

### The effects of morphine on neural activation across several brain regions in naive mice.

**Figure 2:**
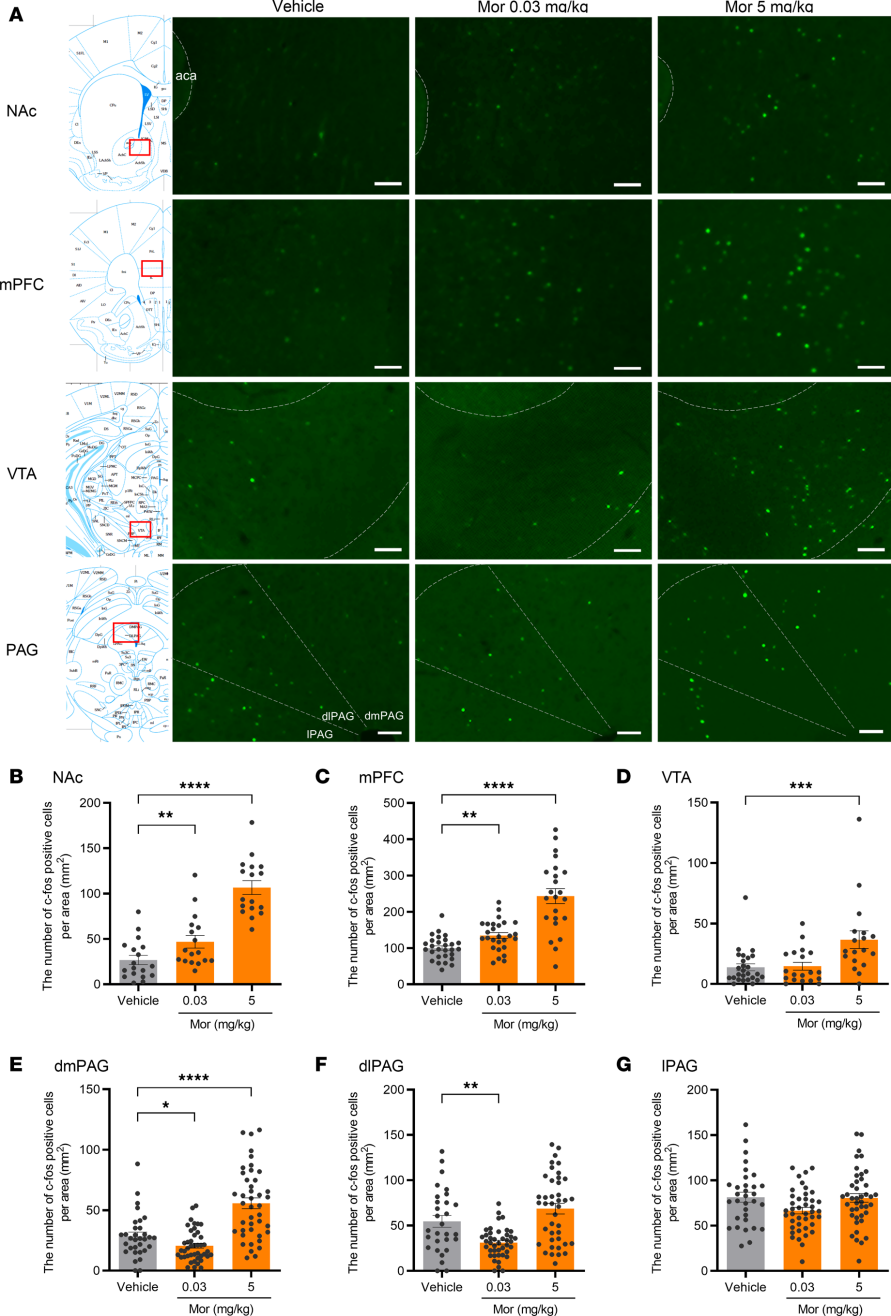
Brain area–specific activation by systemic morphine administration in mice. Morphine (0.03 or 5 mg/kg) or vehicle was s.c. administered to naive C57BL/6J mice 1.5 hours before sampling the brain. (**A**) Representative images of c-Fos–immunostained brain sections of the NAc, mPFC, VTA, and dorsal PAG with the brain atlas map (31). Scale bar: 100 μm. (**B–G**) Quantification of c-Fos–positive cells in each brain region. Results from the NAc (**B**, 17–18 sections/group from 4 mice/group), mPFC (**C**, 23–28 sections/group from 4 mice/group), VTA (**D**, 18–26 sections/group from 4 mice/group), dmPAG (**E**, 32–43 sections/group from 4 mice/group), dlPAG (**F**, 29–42 sections/group from 4 mice/group), and lPAG (**G**, 32–42 sections/group from 4 mice/group) are shown. Data are shown as the mean ± SEM. *P < 0.05, **P < 0.01, ***P < 0.01, ****P < 0.0001, by the parametric tests (**C**, **F**, and **G**) or nonparametric ones (**B**, **D**, and **E**) compared with vehicle-treated mice. Mor, morphine; NAc, nucleus accumbens; mPFC, medial prefrontal cortex; VTA, ventral tegmental area; dmPAG, dorsomedial periaqueductal gray; dlPAG, dorsolateral periaqueductal gray; lPAG, lateral periaqueductal gray.

To identify the brain regions involved in the effects of morphine in promoting social behavior, we quantified neuronal activation in several brain regions using c-Fos immunohistochemistry, which is a marker of neuronal activity. Mice were euthanized 1.5 hours after s.c. administration of morphine (0.03 and 5 mg/kg), and the number of c-Fos–positive cells was quantified using our established automated counting system (Supplemental Figure 1). Initially, we focused on the NAc and medial prefrontal cortex (mPFC) because these regions are known to have a high density of MORs (25–28) and play critical roles in modulating social behavior (10, 29, 30). At the low dose (0.03 mg/kg), we observed a significant increase in the number of c-Fos–positive cells in both the NAc and mPFC (Figure 2, A–C) (31), indicating that, even at such a low dose, morphine could affect neuronal activity in the central nervous system. Consistent with findings in previous reports (32–35), the higher dose of morphine (5 mg/kg) further increased the number of c-Fos–positive cells in both regions (Figure 2, A–C).

Next, we evaluated neuronal activity in the ventral tegmental area (VTA), a brain region associated with addiction and analgesia induced by opioids (36). Although 5 mg/kg morphine, which is analgesic and produces tolerance and dependence with repeated administration (37, 38), significantly increased the number of c-Fos–positive cells in the VTA, 0.03 mg/kg morphine, a prosocial dose, did not have this effect (Figure 2, A and D).

We further investigated the dorsal PAG, which plays critical roles in opioid-induced analgesia and social behaviors (39, 40). Because Franklin et al. reported that activation of the PAG, especially the dorsomedial part (dmPAG), suppressed social interaction behavior (41), we postulated that a high dose of morphine with an analgesic effect would activate the dmPAG and induce an inhibitory effect on social behavior. Expectedly, the dmPAG was activated by administration of 5 mg/kg morphine (Figure 2, A and E), which did not promote social behavior (Figure 1, C and D). In contrast, 0.03 mg/kg morphine did not increase the number of c-Fos–positive cells in any of the 3 subregions (dorsomedial, dorsolateral, or lateral) of the PAG (Figure 2, A and E–G).

### The prosocial effect of low-dose morphine is counteracted by activation of the dmPAG.

**Figure 3:**
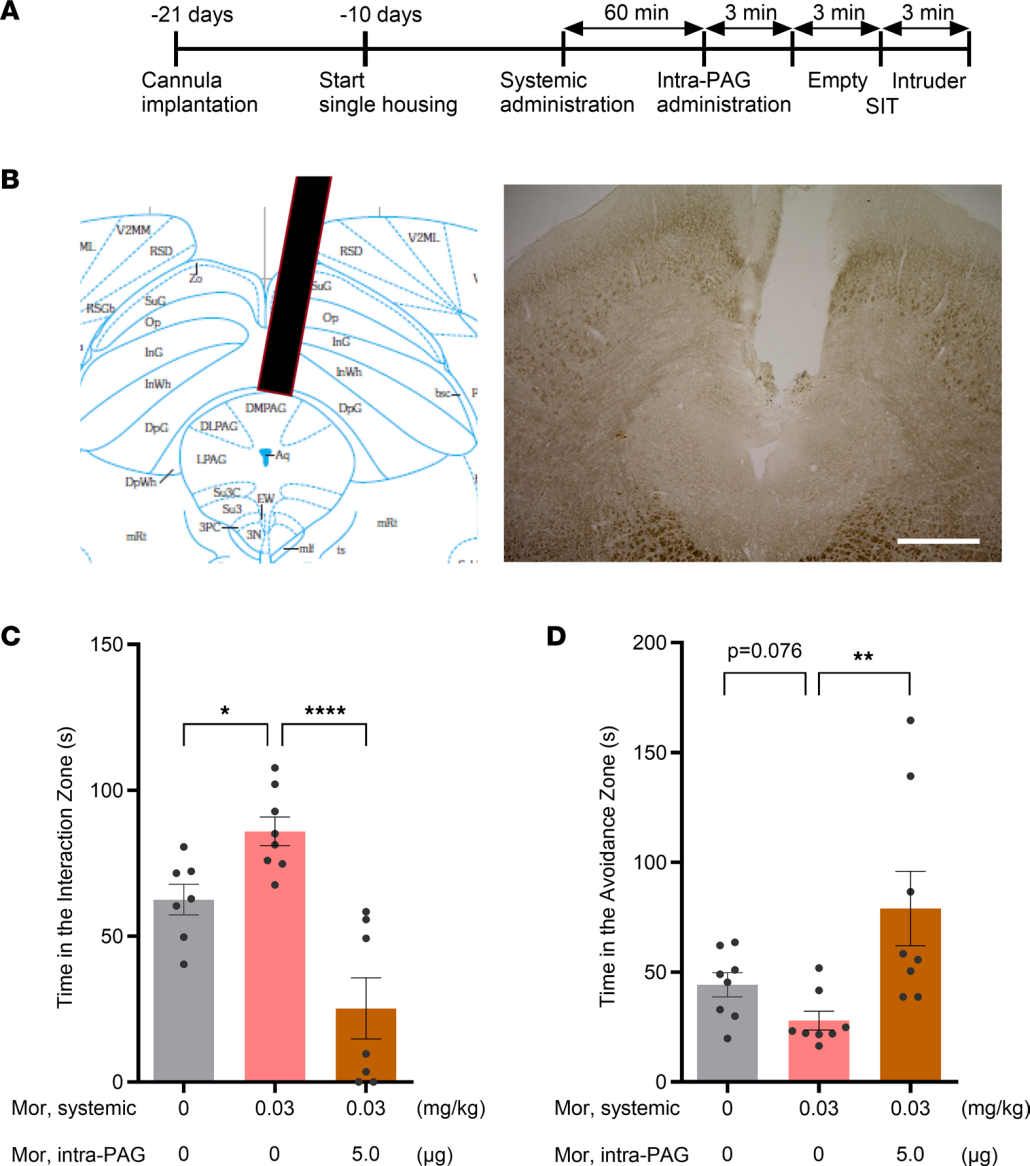
Social behavior induced by morphine was antagonized by topical administration of morphine into the dorsal PAG. (**A**) Timeline of the experiment. (**B**) Schematic of cannula implantation into the dorsomedial part of the PAG, and a representative image of mouse brain sections obtained after the experiment. Scale bar: 500 μm. (**C** and **D**) Effects of intra-PAG administration of morphine on social behaviors in the single-chamber social interaction test (SIT). Single-housed C57BL/6J mice were s.c. injected with morphine (0.03 mg/kg) or vehicle 1 hour before the test. Immediately before the test, morphine (5 μg) or vehicle was infused through the cannula (see Supplemental Methods). The time spent in the interaction zone (**C**) and the avoidance zone (**D**) during a 3-minute test period with an unfamiliar mouse kept in a cage (target session) is shown. *n* = 7–8 animals. *P<0.05, **P < 0.01, ****P < 0.0001, by parametric (**C**) and nonparametric (**D**) tests compared with the group treated with systemic morphine 0.03 mg/kg plus vehicle intra-PAG infusion. PAG, periaqueductal gray; Mor, morphine.

Our c-Fos mapping results led us to hypothesize that, while low-dose morphine selectively activates prosocial brain regions such as the NAc and mPFC, high-dose morphine additionally activates the dmPAG, which negatively regulates social behavior, thereby counteracting the prosocial signals from the activated NAc and mPFC. To examine our hypothesis, we determined whether intra-dmPAG injection of morphine would inhibit the prosocial effect induced by systemic treatment with low-dose morphine. For intra-dmPAG injection, a single cannula was implanted into the dorsal PAG of B6 mice. Following a 3-week recovery period, the SIT was performed after systemic administration accompanied by intra-PAG infusion of drugs (Figure 3, A and B). Systemic administration of low-dose morphine (0.03 mg/kg, s.c.) significantly increased the time spent in the interaction zone when an intruder mouse was presented (Figure 3C; cf. Figure 1A). However, the interaction behavior was significantly decreased by concomitant intra-PAG infusion of 5.0 μg of morphine, which has been reported to induce analgesic effects (42, 43) (Figure 3C). On the other hand, the time spent in the avoidance zone was increased by concomitant intra-dmPAG infusion of morphine (Figure 3D). These results support the idea that stimulation of MORs in the dmPAG, which is induced only by high-dose morphine, counteracts the positive effects of NAc and mPFC activation on social interaction behavior, which underlies the inverted U-shaped dose response of the prosocial effect of morphine.

### The effects of buprenorphine on social behavior in VPA model and Fmr1-KO mice.

**Figure 4:**
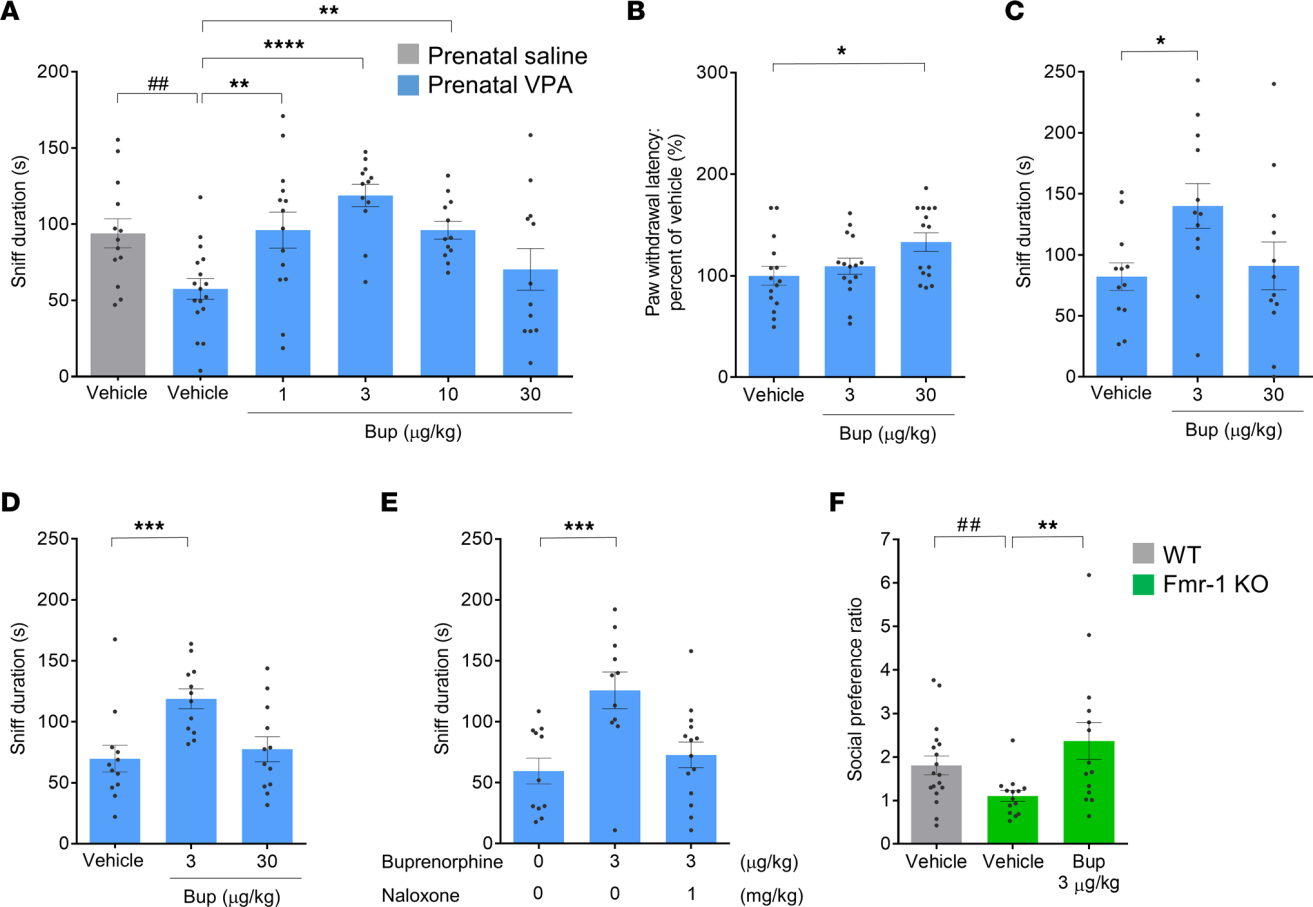
Low-dose buprenorphine increased social behaviors in VPA-treated and *Fmr1*-KO mice. (**A**) Effects of systemic buprenorphine administration on social behavior deficits in mice prenatally exposed to valproic acid (VPA) in the reciprocal social interaction test. Buprenorphine (1, 3, 10, or 30 μg/kg) or vehicle was s.c. injected into male offspring at 8 weeks of age. One hour after administration, the sniffing duration was measured during a 20-minute test period. (**B**) Effects of buprenorphine on thermal nociception in VPA-treated mice. The withdrawal latency at 49°C was measured with a hot-plate test 1 hour after buprenorphine administration (3 or 30 μg/kg, s.c.). (**C** and **D**) Effects of buprenorphine (3 or 30 μg/kg, s.c.) on social interaction deficits in VPA-treated mice were examined at 3 (**C**) or 12 hours (**D**) after administration. (**E**) The effects of buprenorphine (3 μg/kg, s.c.) on social interaction deficits were antagonized by naloxone (1 mg/kg, s.c.), an MOR antagonist, in VPA-treated mice. (**F**) Effects of systemic buprenorphine administration (3 μg/kg, s.c.) on disrupted social preference in *Fmr1*-KO mice. The time spent in the zone with or without an unfamiliar mouse was measured during a 10-minute test period. *n* = 11–18 animals. Data are shown as the mean ± SEM. ^##^*P* < 0.01, by parametric test (**A**) or nonparametric one (**F**) compared with vehicle-treated control mice prenatally exposed to saline (**A**) or vehicle-treated wild-type (C57BL/6J) mice (**F**). *P < 0.05, **P < 0.01, ***P < 0.001, ****P < 0.0001, by parametric tests (**A**–**C**) or nonparametric ones (**D**–**F**) compared with vehicle-treated mice prenatally exposed to VPA (**A–E**) or vehicle-treated *Fmr1*-KO mice (**F**). Bup, buprenorphine; MOR, μ-opioid receptor; *Fmr1*, fragile X mental retardation 1.

Low doses of morphine without analgesic or addictive effects induced prosocial effects, suggesting that maximal stimulation of MORs is unnecessary to promote social behavior, and only partial stimulation of MORs would be sufficient. This finding prompted us to examine the therapeutic potential of buprenorphine, a MOR partial agonist with κ-opioid receptor antagonist potency, in ASD-like model animals. Buprenorphine is a schedule III controlled drug according to Drug Enforcement Administration regulations, indicating its better safety profile and lower abuse liability compared with those of other opioids, such as morphine. Indeed, buprenorphine is broadly used to treat pain and opioid use disorder. Here, we examined the effect of a broad range of buprenorphine doses on social behavior in VPA model mice. We found that at 1 hour after s.c. administration of buprenorphine, reciprocal social interaction deficits of VPA model mice were significantly improved with low doses (1, 3, 10 μg/kg), but not for a higher dose (30 μg/kg), of buprenorphine indicating an inverted U-shaped dose response (Figure 4A). Conversely, in the evaluation of its analgesic effect in the hot-plate test, 30 μg/kg buprenorphine, but not 3 μg/kg, significantly increased the paw-withdrawal latency (Figure 4B), indicating distinct effective doses for promoting social behavior and suppressing pain sensation.

Next, considering the long-lasting efficacy of buprenorphine previously reported (44–46), we assessed the duration of the effects of buprenorphine on social interaction in VPA model mice. Buprenorphine (3 μg/kg, s.c., but not 30 μg/kg) showed sustained effects of improving social behavior at 3 and 12 hours (Figure 4, C and D) as well as at 1 hour after administration (Figure 4A).

To clarify the receptor-mediating prosocial effect of buprenorphine, we examined the effect of naloxone (1 mg/kg, s.c.), a MOR antagonist, in a reciprocal SIT when it was concomitantly injected with buprenorphine (3 μg/kg, s.c.). Cotreatment of naloxone and buprenorphine almost completely blocked the effect of buprenorphine to improve social interaction deficits (Figure 4E). This result suggests that the effect of buprenorphine on social behavior could be mediated mainly by MORs.

Furthermore, to corroborate the therapeutic potential of buprenorphine on social behavior deficits, we performed an efficacy study using *Fmr1*-KO mice, a well-established animal model of fragile X syndrome. Many individuals with fragile X syndrome show ASD-like social behavior impairments, some of which are recapitulated in *Fmr1*-KO mice (47). Since *Fmr1*-KO mice showed the impairments only in a social novelty session but not in a sociability session in the 3-chamber test in the previous study (47), we evaluated the effect of buprenorphine on social novelty behaviors of *Fmr1*-KO mice. After s.c. administration of 3 μg/kg buprenorphine, social behavior impairment in *Fmr1*-KO mice was significantly improved (Figure 4F), whereas exploratory behavior around the cages was not altered (Supplemental Figure 4). This result strengthens the notion that buprenorphine has the therapeutic potential to ameliorate social behavioral deficits in ASD.

### The effects of buprenorphine on neural activation across several brain regions in VPA model mice.

**Figure 5:**
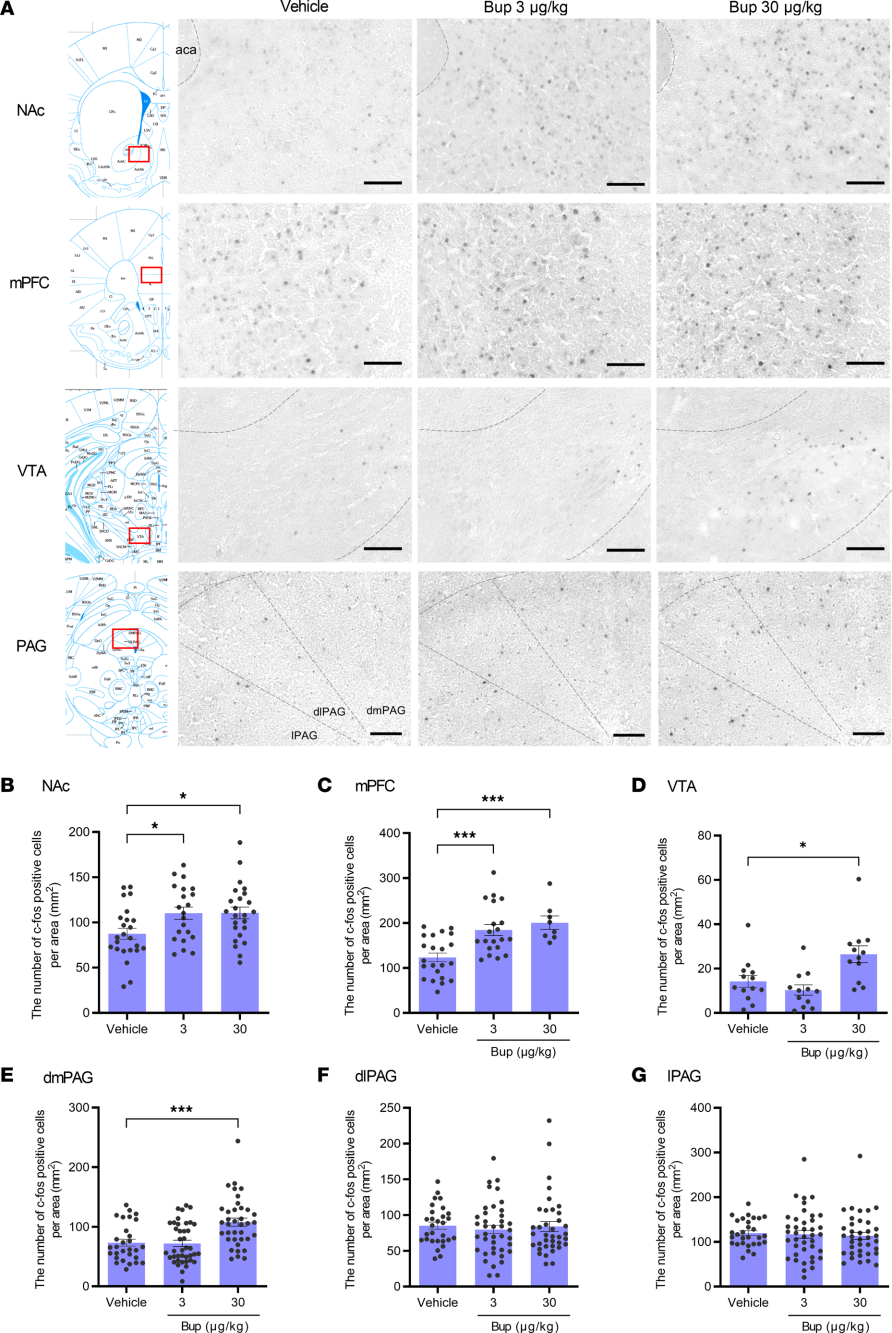
Brain area–specific activation by systemic buprenorphine administration in VPA-treated mice. Male offspring born to mothers injected with valproic acid (500 mg/kg, intraperitoneal) were subjected to immunohistochemical analysis at 8 weeks of age. Buprenorphine (3 or 30 μg/kg) or vehicle was s.c. administered 1.5 hours before sampling the brain. (**A**) Representative images of c-Fos–immunostained brain sections containing the NAc, mPFC, VTA, and dorsal PAG. Scale bar: 100 μm. (**B–G**) Quantification of c-Fos–positive cells in each brain region. The results in the NAc (**B**, 21–24 sections/group from 4 mice/group), mPFC (**C**, 8–22 sections/group from 3–7 mice/group), VTA (**D**, 12–13 sections/group from 2 mice/group), dmPAG (**E**, 29–40 sections/group from 6 mice/group), dlPAG (**F**, 29–40 sections/group from 6 mice/group), and lPAG (**G**, 29–40 sections/group from 6 mice/group) are shown. Data are shown as the mean ± SEM. *P < 0.05, ***P < 0.001, by parametric (**B** and **D**) or nonparametric (**C** and **E**–**G**) tests compared with vehicle-treated mice. Bup, buprenorphine; NAc, nucleus accumbens, mPFC, medial prefrontal cortex; VTA, ventral tegmental area; dmPAG, dorsomedial periaqueductal gray; dlPAG, dorsolateral periaqueductal gray; lPAG, lateral periaqueductal gray.

To identify the brain regions involved in the effect of buprenorphine on social behavioral deficits, we performed c-Fos immunohistochemistry, as described above. Mice were euthanized 1.5 hours after s.c. administration of buprenorphine (3 and 30 μg/kg), and the number of c-Fos–positive cells was quantified. Buprenorphine at a dose of 3 μg/kg, which improved social interaction deficits, significantly increased the number of c-Fos–positive cells in both the NAc (Figure 5, A and B) and mPFC (Figure 5, A and C) but not in the VTA (Figure 5, A and D) or in 3 subregions (dorsomedial, dorsolateral, and lateral) of the PAG (Figure 5, A and E–G). Buprenorphine, at a dose of 30 μg/kg, which showed an analgesic effect (Figure 4B) (44) but not a prosocial effect (Figure 4, A, C, and D), significantly increased the number of c-Fos–positive cells in the NAc, mPFC, VTA, and dmPAG. The relationship between the effect on social behavior and the pattern of brain activation was consistent between morphine and buprenorphine. These results further suggest that the dmPAG determines the effects of MOR agonists on social behavior, in addition to activation of the NAc and mPFC.

## Discussion

In the present study, we investigated the effects of MOR agonists on sociability impairments as observed in ASD from a nonclinical perspective. Low doses of both morphine and buprenorphine enhanced or improved sociability without exhibiting analgesic effects. At those doses, activation of the NAc and mPFC was observed with both drugs, implying the involvement of these brain regions in facilitating social behavior. Conversely, at analgesic doses, neither of the drugs enhanced or improved social behaviors. Activation of the dmPAG, in addition to the NAc and mPFC, was induced by both drugs at those doses, indicating a potential role of the dmPAG in inhibiting social behaviors, as reported in a previous study (41). Indeed, when morphine was injected into the dmPAG, the enhancement in sociability induced by systemic administration of low-dose morphine was antagonized. These results suggest the important role of brain region–specific neural activation by low-dose opioids in promoting social behaviors.

Previous studies reported that activation of MORs by administration of their agonists at analgesic doses reduced social investigatory behavior (11, 48, 49) and responses to social play (11). Conversely, attenuation of MOR signaling also has a negative effect on sociability, considering that social interaction behavior is decreased in *Oprm1*-KO mice (7) and animals treated with MOR antagonists (10, 49–51). Based on these findings, it has been hypothesized that maintaining an optimal activation state of the MORs is important in driving social behavior (6). In the present study, we demonstrated that morphine and buprenorphine improved sociability with doses lower than the analgesic doses in naive and ASD-like mice, indicating that an optimally activated state of MOR signaling for sociability could be achieved by administering nonanalgesic low-dose opioids in both naive and ASD-like conditions.

As previously mentioned, it has been suggested that, while excessive activation of MORs suppresses social behavior, appropriate activation of MORs promotes this behavior. The mechanism underlying such a relationship between MOR activation and social behavior, however, remains to be elucidated. In this study, we found that prosocial doses of morphine and buprenorphine induced neural activation in the NAc and mPFC but not in the dmPAG. In a previous study, local injection of a MOR agonist into the NAc enhanced social behavior, suggesting that MOR activation in the NAc may be sufficient to promote social behavior (10). The NAc of both humans and rodents has a high density of MORs (25, 28), indicating that the NAc is an important site of action of MOR agonists. In addition, [^35^S]GTPγS binding stimulated by a MOR agonist was 2–3 times higher in the NAc than that in the PAG and VTA, suggesting a higher expression level of functional MORs in the NAc (27). Likewise, the mPFC is also suggested to play a crucial role in the control of social behavior (29, 30, 52), and the MOR expression level in the PFC is higher than that in other cortical regions (25, 26). Thus, the higher density of MORs in the NAc and mPFC would facilitate formation of an agonist and receptor complex and transduce signaling, even at low agonist concentrations, leading to higher sensitivity of the NAc and mPFC to MOR agonists than that in the PAG and VTA.

The role of the PAG in social behavior has been investigated and its activation has been reported to induce anxiety, avoidance, and defensive behaviors (39, 40). In particular, Franklin et. al. directly demonstrated, through chemogenetic manipulation followed by c-Fos analysis, that neural activation in the dmPAG negatively controls social behaviors (41). Taken together with our results, high-dose, but not low-dose, opioids can induce neural activation in the dmPAG and thereby counteract the prosocial signals caused by activation of the NAc and mPFC, which would underlie the inverted U-shaped dose-dependent effects of morphine and buprenorphine on social behavior. We, however, cannot exclude the involvement of other brain regions reported to be activated by analgesic opioid doses (33–35), and further investigation is necessary to comprehensively elucidate the neural circuit underlying the control of social behavior by opioids.

When opioids are used for therapeutic purposes, caution should be exercised regarding their dependence and abuse liabilities, particularly when considering the opioid crisis in the US and its societal problems (53). The reward circuitry, primarily involving dopaminergic neurons in the VTA, plays a central role in the development of opioid dependence (36). In this study, we showed that opioids could improve social behavior at doses that did not increase neural activation in the VTA (Figures 2 and 5). Consistently, prosocial doses of morphine did not induce conditioned place preference in a previous study (54). Thus, the risk of dependence when prosocial doses of opioids are used is expected to be low, which needs to be cautiously examined in clinical trial. Furthermore, buprenorphine is categorized as a schedule III controlled drug, similar to methylphenidate, which is widely used to treat attention deficit hyperactivity disorder, a developmental disorder. On the basis of these findings, low-dose buprenorphine could be a viable option to treat the pediatric population with ASD.

Regarding the therapeutic potential for ASD, low-dose opioids, especially buprenorphine, have several advantages compared with drug candidates that were highly expected but failed to meet the endpoint in clinical trials, such as oxytocin, balovaptan, and bumetanide. First, the opioid system is highly conserved among species, as exemplified by the analgesic effect commonly observed in humans and rodents, implying that our preclinical findings regarding the role of opioids in social behavior could be translated into clinical results. In fact, MOR activation in the NAc was correlated with an increased desire for social interaction (55), and specifically, low-dose buprenorphine was reported to enhance positive responses to social stimuli (55), which are consistent with our findings in mice. In addition, buprenorphine was reported to improve depressive symptoms and suicidal ideation, supporting the potential of buprenorphine to treat psychiatric disorders involving motivational deficits (56, 57). Second, opioids showed positive effects on social behavior in multiple types of mice, including naive, VPA-treated, and *Fmr1*-KO mice. Combined with the clinical results mentioned above, buprenorphine is expected to be efficacious for a wide range of heterogeneous individuals with ASD. Finally, buprenorphine showed a sustained effect in promoting social behavior (Figure 4, C and D), which is in contrast to the short duration of efficacy of oxytocin in the VPA model (58). This finding could be attributed to the slow dissociation of buprenorphine from MORs (59, 60). In addition, prolonged exposure of humans to buprenorphine further extended the duration of the therapeutic effect to longer than 28 hours (46). Thus, the receptor binding and pharmacokinetic characteristics of buprenorphine could be suitable to facilitate sociability throughout the day, which would be critical to improve social behavior deficits in individuals with ASD during a reasonable time frame. Taken together, the findings indicate that buprenorphine has great potential to treat sociability deficits and achieve an acceptable safety profile as an ASD therapy. On the basis of these results and a case report of 1 patient with ASD taking buprenorphine, implying its efficacy for ASD symptoms (61), clinical testing of the efficacy and safety of buprenorphine in ASD is warranted.

In conclusion, low doses of MOR agonists promoted social behaviors without inducing analgesic effects in naive and ASD-like model animals by activating the NAc and mPFC. These effects on social behaviors were diminished at higher analgesic doses for which the dmPAG was activated. Thus, we clearly show dose-dependent differences in the pharmacological effects of opioids on social behaviors and provide evidence that brain region–specific neural activation is a key factor for opioids to appropriately promote these behaviors. This study sheds light on drug development for sociability impairments in psychiatric disorders and specifically suggests that low-dose MOR agonists could be promising treatments for sociability symptoms in ASD.

## Methods

### Sex as a biologic variable.

Our study examined male mice, because ASD is more common in male than female individuals and male animals exhibited less variability in behavioral assays.

### Animals.

Experimental animals were purchased from Nihon CLEA (male B6 mice) and from Japan SLC Inc. (ICR [CD-1] mice). Animals were housed on a standard 12-hour light/dark cycle and given food and water ad libitum. Mice were housed in groups of 3–4 animals unless otherwise noted.

### Drug treatment.

Morphine was purchased from Takeda Pharmaceuticals. Buprenorphine was purchased from Otsuka Pharmaceutical Co. Ltd. Unless otherwise stated, both drugs were dissolved in 0.9% NaCl solution (saline, Otsuka Pharmaceutical Co. Ltd.) and s.c. administrated at a dose of 10 mL/kg.

### Preparation of a VPA-induced ASD-like mouse model.

Mouse model development was conducted as previously reported (13, 58, 62). In brief, pregnant CD-1 mice were randomly divided into 2 groups and intraperitoneally administered either 500 mg/kg VPA (Sigma-Aldrich) or saline on embryonic day 12.5. VPA was dissolved in saline (Otsuka Pharmaceutical Co. Ltd.), and the volume of injection was 10 mL/kg. Offspring born to VPA- and saline-treated mothers were housed in groups of 5–6 mice of the same sex on postnatal day 21. Only male offspring were subjected to experiments at 8 weeks of age.

### Single-chamber SIT.

Animals were housed individually for 10 days before the test and were habituated to the interaction arena (42 cm width × 42 cm depth × 42 cm height) for 15 minutes on the day before the test. The test was performed as previously reported with minor modifications (63, 64). On the test day, test mice were placed in an arena with an empty wire cage (10 cm width × 6.5 cm depth × 42 cm height). The animals were given 3 minutes to explore the arena (empty session) and then removed. A novel B6 mouse (male, 1 week younger than the test mouse) enclosed in a wire cage was placed in the arena, and the same procedure as that used in the empty session was repeated (target session). The time spent in the area surrounding the wire cage (interaction zone, 24 cm × 14 cm) and in the corner zones (9 cm × 9 cm) opposite to the interaction zone were measured as the social interaction time and avoidance time, respectively, using an ANY-maze Video Tracking System (Stoelting Co.).

### Reciprocal SIT.

The test was conducted as previously reported (13, 65). In brief, test mice (resident mice) were individually habituated to the experimental cage for 60 minutes. Then, an intruder mouse (male CD-1, 1 week younger than the test mouse) was introduced into the cage. The sniffing behaviors (face and anogenital sniffs) of the resident mouse were measured over the total experimental period of 20 minutes.

### Three-chamber social preference test.

The test was conducted as previously reported with minor modifications (66). In brief, under an illumination of 5 lx, test mice [10- to 12-week-old male B6 or *Fmr1* KO (B6.129P2-Fmr1tm1Cgr/J, JAX, stock no. 003025)] were placed in the central chamber of the social interaction apparatus (67), which was composed of a clear Plexiglas box (41 cm × 60 cm × 23.5 cm) divided into 3 interconnected chambers. The test mouse was given the choice to interact with either a wire cup with a familiar cage mate (located in 1 side chamber) or a similar wire cup containing an unfamiliar male B6 mouse (male, 3 weeks younger than the test mouse, located in the opposite chamber). The amount of time the test mouse spent in each of the 3 chambers and in the area surrounding each cup was measured during a 10-minute trial using EthoVision XT (Noldus Information Technology).

### Statistics.

All statistical analyses were carried out using GraphPad Prism 6 and python scikit-posthocs package. Normality of the data distribution was tested using Shapiro-Wilk’s test. The data with Gaussian distribution was tested using parametric methods such as 2-tailed unpaired *t* test, Welch’s test when comparing 2 groups with the different standard deviations, or 1-way analysis of variance followed by Holm-Šidák’s multiple comparisons test. The data not following normal distribution was tested using nonparametric Mann-Whitney’s test or Kruskal-Wallis test followed by Conover’s multiple comparison test. In all cases, the comparisons were considered statistically significant at *P* < 0.05. All data are presented as the mean ± SEM.

### Study approval.

All animal procedures were approved by the Institutional Animal Care and Use Committees of Osaka University, Hiroshima University, and Shionogi & Co. Ltd. and were performed in accordance with the Guidelines for Animal Care and Use of Osaka University, Hiroshima University, and Shionogi & Co. Ltd.

### Data availability.

All data needed to evaluate the conclusions of the manuscript are present in the paper and/or supplemental materials. Values for graphs in the figures and supplemental figures are provided in the Supporting Data Values file.

## Author contributions

AN, HY, and Y Ago conceptualized and designed the study. SO, MN, HK, MH, SH, K Takasu, RK, Y Azuma, RY, TK, EI, TT, and Y Ago conducted the experiments. NO established the automated counting system of c-Fos–positive cells. SO, MN, HY, and Y Ago constructed the graphs. K Takuma and Y Ago provided the reagents. YK, SA, AN, KO, K Takuma, HH, HY, and Y Ago supervised the study. SO and HY wrote the manuscript. SO, HY, and Y Ago reviewed and edited the manuscript. SO was assigned as the first co–first author because of his contribution to writing.

## Supplementary Material

Supplemental data

Supporting data values

## Version 1 12/06/2024

Electronic publication
