# Supplementary material for: Brain region–specific neural activation by low-dose opioid promotes social behavior
Source: JCI Insight. 2024 Dec 6;9(23):e182060. doi: 10.1172/jci.insight.182060 (PMC11623950; doi:10.1172/jci.insight.182060)
Supplement: Supplemental data [file jciinsight-9-182060-s208.pdf]

Supplemental Material for

· Brain region-specific neural activation by low-dose opioid promotes social behavior

Soichiro Ohnami *et al.*

*Corresponding author. Hidekuni Yamakawa, Yukio Ago

Email: hidekuni.yamakawa@pingan-shionogi.com (H.Y.); yukioago@hiroshima-u.ac.jp (Y. Ago)

This file includes:

- Figure S1-4
- Supplemental Materials and Methods

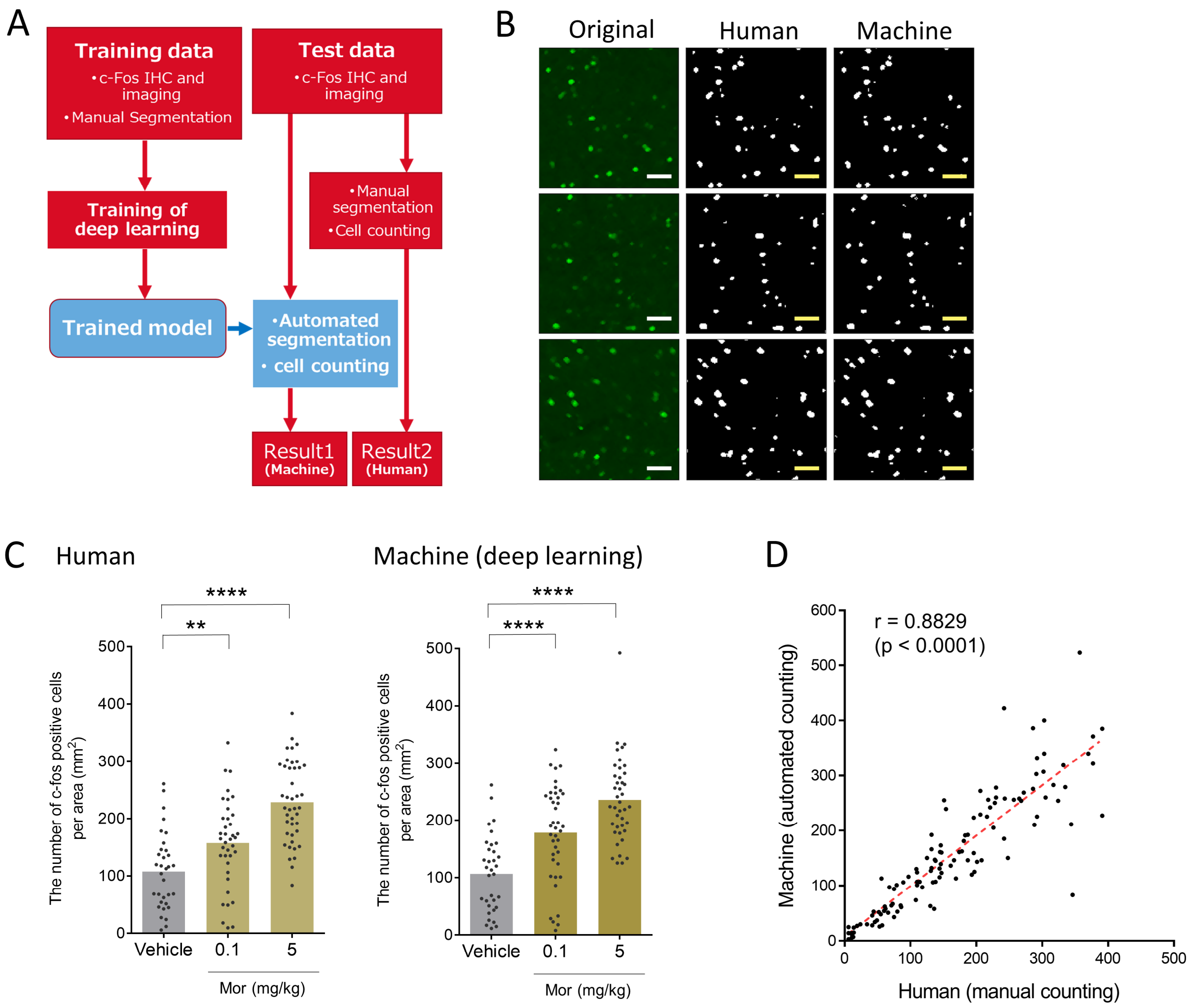

Figure S1. Automated counting of c-Fos-positive cells

(A) Scheme for automated counting. The brain regions of interest was segmented manually from c-Fos IHC images. Using these images and those manually binarized data, a trained model was created with machine learning. Segmentation was also performed on another c-Fos IHC images (Test data) different from the training data. The results of automated counting using the trained model were compared with manual counting. (B) Examples of binalization from automated counting using the trained model and manual counting. Scale bar, 25 µm. (C, D) The validation of the automated counting method. Vehicle, morphine 0.1 mg/kg, and morphine 5 mg/kg were subcutaneously administered to wild-type B6 mice, and c-Fos-positive cells in the mPFC were counted. Values in (C) indicate the mean ± SEM (n = 5–6 animals/group). The results obtained using manual counting by a human analyzer and automated counting with ML were highly correlated (r = 0.8829) and statistically significant ($p < 0.0001$). **$P < 0.01$, ****$P < 0.0001$, compared with mice with s.c. administration of vehicle.

ROI: region of interest, IHC: immunohistochemistry, mPFC: medial prefrontal cortex, Mor: morphine, r: Pearson's correlation coefficient.

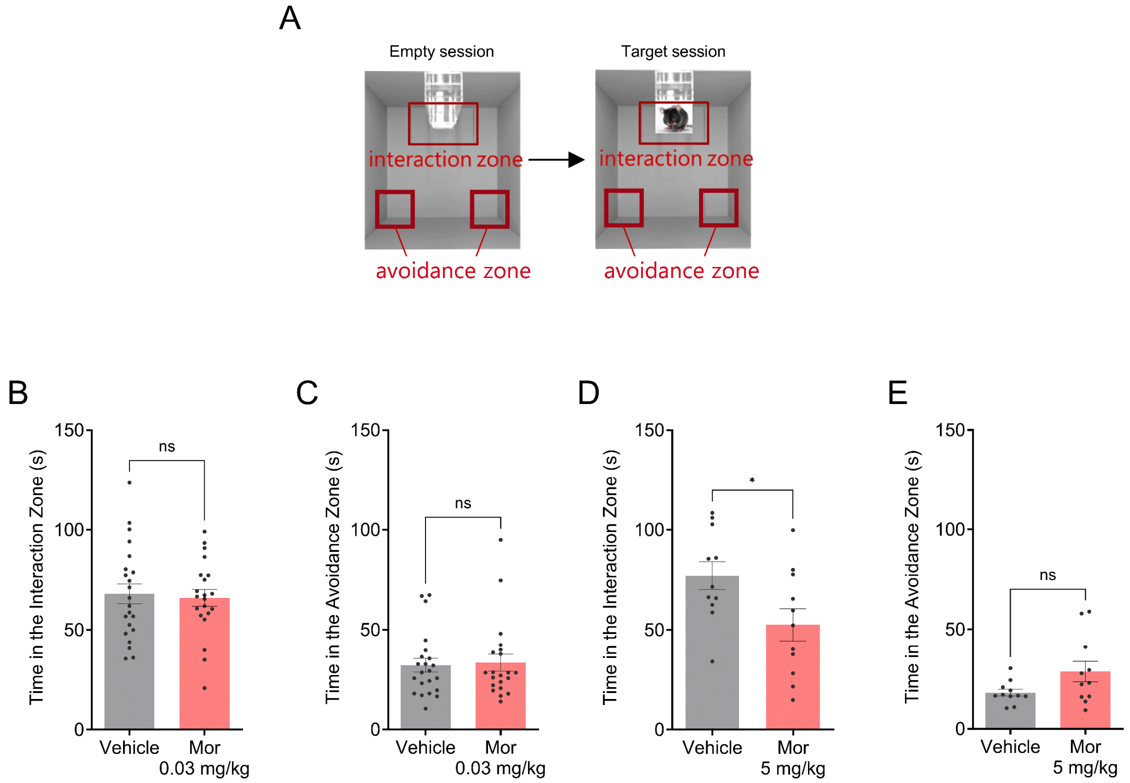

Figure S2. Single-chamber SIT

(A) Upper view of SIT box. The times staying "interaction zone" and "avoidance zone" were quantified in empty session (B-E) and target session (Figure 1). The results of mice treated with 0.03 mg/kg Mor are B (interaction zone) and C (avoidance zone), those with 5 mg/kg Mor are D (interaction zone) and E (avoidance zone). Values indicate the mean ± SEM. ns, not significant, **P* < 0.05 by parametric tests (B, D, E) or a non-parametric one (C) compared with vehicle-treated mice. Mor; morphine.

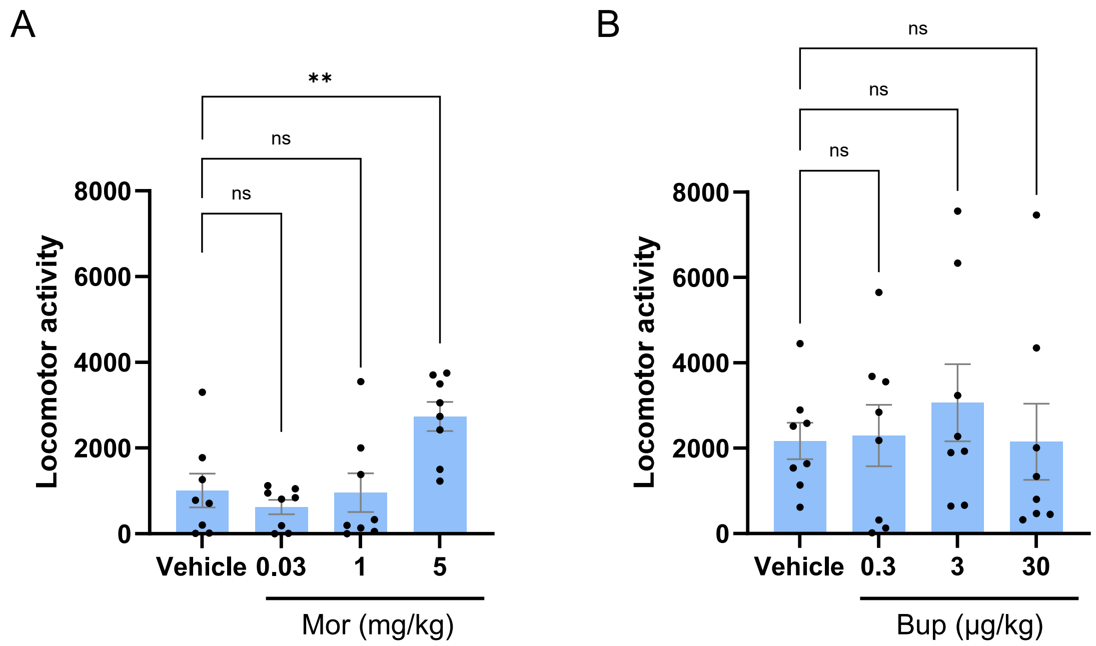

Figure S3. Locomotor activity after treatment with morphine or buprenorphine

The locomotor activity of CD-1 mouse was measured for 50 min from 10 min after the injections of morphine (A) and buprenorphine (B). Values indicate the mean ± SEM. ns, not significant, $^{**}P < 0.01$ by a non-parametric test compared with vehicle-treated mice. Mor; morphine, Bup; buprenorphine.

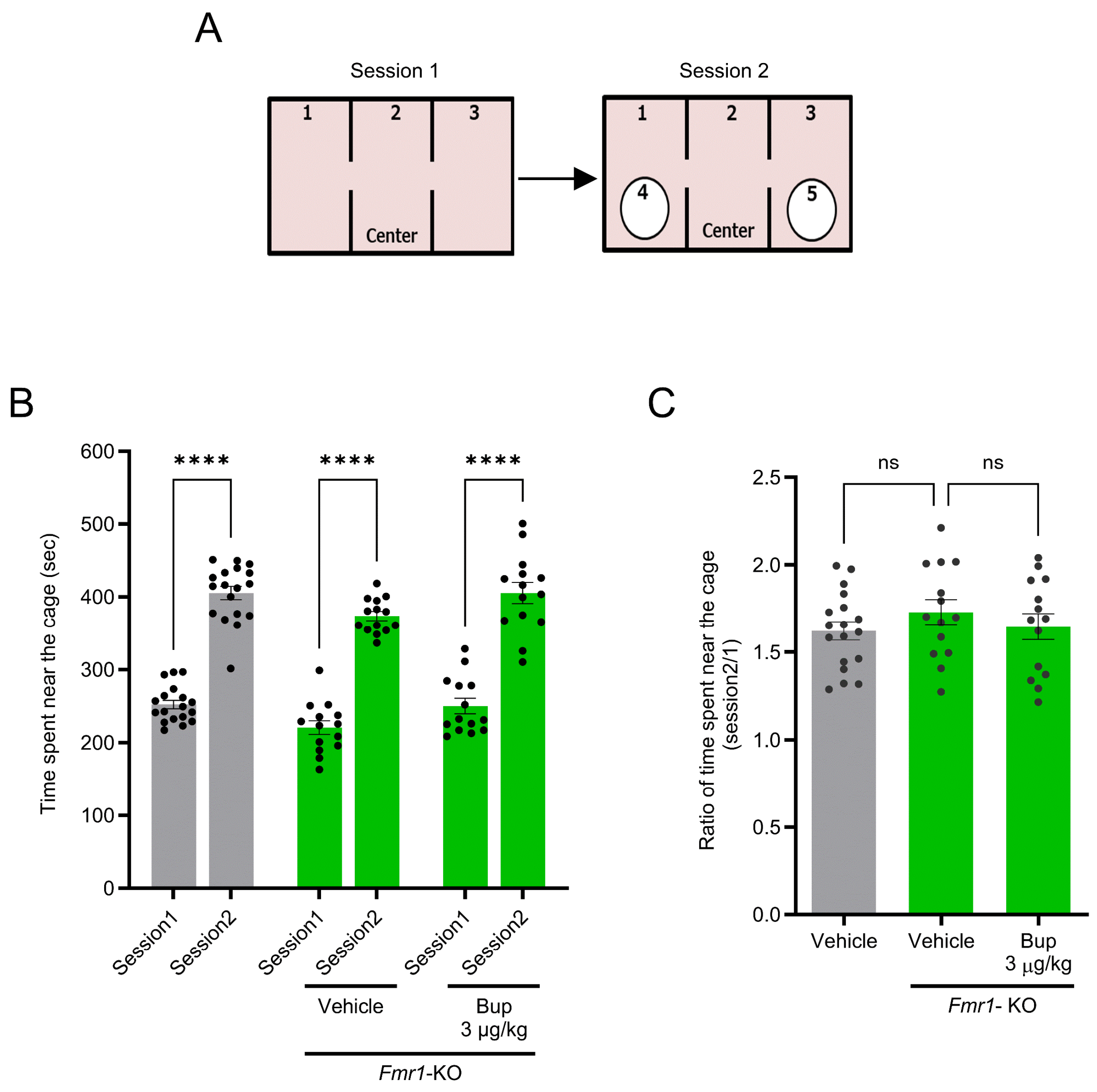

Figure S4. Three-chamber test

(A) Schematic of three chamber test. (B) The times spent near the cages were quantified in sessions 1 and 2. (C) The ratios of time spent near the cages in sessions 1 and 2 were individually calculated to compare the attention to the object (cage) between the groups. Values indicate the mean ± SEM. ns, not significant, ****P < 0.0001 by parametric tests in the comparisons between sessions 1 and 2 (B) or vs vehicle-treated *Fmr1*-KO group. Bup; buprenorphine.

Supplemental Materials and Methods

Locomotor activity test

The locomotor activity of mice was measured using a digital counter system with an infrared sensor (Supermex®, Muromachi Kikai Co., Ltd., Tokyo, Japan) (1). The CD-1 mice were placed individually in a novel clear plastic cage (28 cm × 17 cm × 12 cm). After a 60-min habituation period, mice were injected with morphine, buprenorphine, or saline, and then locomotor activity was analyzed for 50 min (interval: 10 min) from 10 min after the drug injection.

Hot plate test

The test was conducted as previously reported (2). In brief, the mice were individually placed on a hot metal plate with the temperature maintained at 49 ± 0.5 °C (#35100; Ugo Basile, Gemonio, Italy). The latency until the first sign of hindpaw licking or jumping was recorded as an indicator of the nociceptive threshold. The cutoff time was set at 60 s to avoid injury to the paws.

Microinjection

For the cannula implantation surgery, mice were anesthetized with isoflurane (Forane, Abbott Japan LLC, Tokyo, Japan; 3%–4% initially, maintained at 1%–2%) using an MK-A110 (Muromachi Kikai, Tokyo, Japan). Petroleum jelly (Taiyo Seiyaku, Tokyo, Japan) was applied to the eyes to prevent drying. The mice were positioned in a stereotaxic instrument (SR-6R-H, Narishige, Tokyo, Japan), and the head was stabilized using ear bars and a mouth bar. The skull was exposed, and holes were drilled above the target region. A single guide cannula with a dummy cannula (RWD-62064 and RWD-62164, respectively; RWD Life Science, San Diego, CA) was implanted into the dorsal PAG (anteroposterior: −4.0 mm from bregma, mediolateral: ±0.4–0.5 mm from bregma, dorsoventral: −2.2 mm from the dura with an angle of 12°), and the cannula was secured with dental cement (Self-Curing Acrylic Resin, Sun Medical, Shiga, Japan or Shofu, Kyoto, Japan). The mice were allowed to recover for at least 3 weeks after surgery.

For intra-PAG infusion, mice with cannula implantation underwent habituation to the infusion procedure once a day for 3 days. The mice were restrained, and the implanted cannula was connected to an injection cannula (RWD-62264, RWD Life Science, length: 6.2 mm) using polyethylene tubing (427411, BD Intramedic, Franklin Lakes, NJ). Then, the mice were allowed

to freely move in their home cage for 3 min, and the injection cannula was disconnected. On the test day, morphine (Takeda Pharmaceuticals, Tokyo, Japan) was dissolved in sterilized phosphate-buffered saline (PBS) (11480-35, Nacalai Tesque, Kyoto, Japan). Immediately before the SIT, morphine was delivered into the PAG using a syringe pump. The drugs were infused at a rate of 0.1 μl/min in a volume of 0.5 μl using Hamilton syringes (84853, Hamilton, Reno, NV) and a syringe pump (LEGATO 180, KD Scientific, Holliston, MA). The correct placement of the cannula was confirmed by histological analysis of coronal brain slices. Mice with inaccurately placed cannulas were excluded from the study.

Histology, immunohistochemistry, and imaging

For the histological analysis of naïve B6 mice, animals were deeply anaesthetized with isoflurane and transcardially perfused with cold 4% paraformaldehyde perfusion fixation solution (09154-85, Nacalai Tesque). Brains were extracted and post-fixed in the fixation solution for 24 hours at 4 °C and then transferred to 30% sucrose in PBS for cryoprotection for ≥ 3 days at 4 °C. The brains were then frozen, and 20-μm coronal sections were cut using a cryostat (CM1860, Leica Microsystems, Wetzlar, Germany). Free-floating sections were incubated with blocking solution (5% normal goat serum (S-1000, Vector Laboratories, Newark, CA) and 0.3% Triton X-100 (35501-02, Nacalai Tesque) in PBS) for 0.5 hours. Primary antibodies against Fos (1:1000, catalog no. 2250, Cell Signaling Technology, Danvers, MA) were diluted in blocking solution, and the sections were incubated overnight at 4 °C with gentle shaking. The sections were then washed three times in PBS and incubated with secondary antibodies (Alexa Fluor 488-conjugated anti-rabbit IgG, 1:1000, or Alexa Fluor 594-conjugated anti-rabbit IgG, 1:1000, Thermo Fisher Scientific, A11034 or A11037) in 0.3% Triton X-100 in PBS for 2 hours at room temperature. The sections were then washed in PBS and stained with DAPI (340-07971, Dojindo, Kumamoto, Japan) for more than 3 min. After the final washes in PBS, the sections were mounted in FluorSave (catalog no. 345789, Merck Millipore, Burlington, MA). Images were acquired using a fluorescence microscope (BZ-X, Keyence, Osaka, Japan).

For the histological analysis of VPA model mice, 1.5 hours after buprenorphine administration, the whole brain was removed under isoflurane anesthesia and immediately frozen on dry ice. Coronal sections of 20-μm thickness were cut using a cryostat, mounted onto glass slides, and air-dried. Prior to staining, the tissue was fixed by immersing it in 4%

paraformaldehyde/0.1 M PBS for 10 min. Antigen retrieval was performed by heating the sections in Target Retrieval Solution (DAKO, Holstebro, Denmark) for 40 min. Endogenous peroxidase was inactivated by immersing the sections in 0.3% H_2O_2 (in PBS) for 20 min at room temperature. The sections were then incubated in a blocking solution (10% normal horse serum (Vector Laboratories) and 0.3% Triton X-100 in PBS) for 0.5 hours. Then, the sections were incubated with anti-c-Fos rabbit polyclonal primary antibodies (diluted 1:1000 in the blocking solution; sc-271243, Santa Cruz Biotechnology, Santa Cruz, CA) at 4 °C for 48 hours. After washing the sections three times with PBS, they were incubated with a biotinylated anti-mouse IgG antibody (Vectastain ABC kit, Vector Laboratories) for 0.5 hours at room temperature, followed by reaction with 3,3'-diaminobenzidine for 1.5 min. The sections were coverslipped after washing three times with PBS. Bright-field images were acquired using a microscope (BZ-X, Keyence). ImageJ software was used to randomly select six points in the background, and the threshold for positive cells was set to the mean grey value of the background minus 5 standard deviations. Eight or more pixels were counted and used to calculate the number of cells per mm^2.

Automatic counting of c-Fos-positive cells

We used machine learning-based image analysis for automated and objective cell counting. The architecture design was based on U-Net, which is a popular type of deep learning model used for segmentation in the medical field (3). The number of depth layers was set to six during the encoding and decoding processes. A residual block was also introduced into this model to address the problem of vanishing gradients (4, 5). The slope coefficient of the leaky rectified linear unit was set to 0.2. The model was trained for 100 epochs with Dice loss (1 – Dice coefficient) as the loss function using the Adam optimizer (6). The initial learning rate was set to 1.0×10^{-3} and decayed in steps to 1.0×10^{-4} at 40 epochs and to 1.0×10^{-5} at 80 epochs. The learning process included online augmentation by applying the following transformations: translation to the left, right, up, and down within a maximum width of 10%; rotation from −10° to 10°; scaling between −10% and 10% of the image size. The size of the training dataset was increased 50-fold. The model was trained with the external dataset (microscopic images of c-Fos immunohistochemistry from 40 slices) with manual annotation of positive cells. The microscopic images were divided into 128 × 128 pixels with 64 strides, with 80% of the data used for training and the remaining 20% for internal validation. The model was constructed using TensorFlow and Keras. After the model was

derived, the external validation data were applied for automated segmentation of c-Fos-positive cells using the model (Supplemental Figure S1). The segmented images were converted into binary images with a threshold value of 0.99 and automatically contour-extracted using the contourArea function in OpenCV (https://opencv.org/). Regions with a circularity of 0.5 or greater were extracted as positive cells, and the number of cells was automatically measured. This method was used to evaluate the effect of morphine (Figure 2) and reduced the analysis time to approximately 1/10 of that of conventional manual analysis by humans.

References

1. Yokoyama R, et al. (S)-norketamine and (2S,6S)-hydroxynorketamine exert potent antidepressant-like effects in a chronic corticosterone-induced mouse model of depression. *Pharmacol Biochem Behav*. 2020;191:172876.
2. Horiguchi N, et al. Involvement of spinal 5-HT1A receptors in isolation rearing-induced hypoalgesia in mice. *Psychopharmacology (Berl)*. 2013;227(2):251–261.
3. Ronneberger O, Fischer P, Brox T. U-Net: Convolutional Networks for Biomedical Image Segmentation. 2015.
4. Zhang Z, Liu Q, Wang Y. Road Extraction by Deep Residual U-Net. *IEEE Geoscience and Remote Sensing Letters*. 2018;15(5):749–753.
5. Nishi H, et al. Deep Learning–Derived High-Level Neuroimaging Features Predict Clinical Outcomes for Large Vessel Occlusion. *Stroke*. 2020;51(5):1484–1492.
6. Kingma DP, Ba J. Adam: A Method for Stochastic Optimization. 2014.
